# The Combination of CD8αα and Peptide-MHC-I in a Face-to-Face Mode Promotes Chicken γδT Cells Response

**DOI:** 10.3389/fimmu.2020.605085

**Published:** 2020-11-23

**Authors:** Yanjie Liu, Rong Chen, Ruiying Liang, Beibei Sun, Yanan Wu, Lijie Zhang, Jim Kaufman, Chun Xia

**Affiliations:** ^1^ Department of Microbiology and Immunology, College of Veterinary Medicine, China Agricultural University, Beijing, China; ^2^ Key Laboratory for Insect-Pollinator Biology of the Ministry of Agriculture, Institute of Apicultural Research, Chinese Academy of Agricultural Sciences, Beijing, China; ^3^ Department of Pathology, University of Cambridge, Cambridge, United Kingdom; ^4^ Department of Veterinary Medicine, University of Cambridge, Cambridge, United Kingdom

**Keywords:** chicken γδT cells response, CD8/pMHC-I interaction, face-to-face mode, cCD8αα/pBF2*1501, cCD8αα/pBF2*0401

## Abstract

The CD8αα homodimer is crucial to both thymic T cell selection and the antigen recognition of cytotoxic T cells. The CD8-pMHC-I interaction can enhance CTL immunity *via* stabilizing the TCR-pMHC-I interaction and optimizing the cross-reactivity and Ag sensitivity of CD8^+^ T cells at various stages of development. To date, only human and mouse CD8-pMHC-I complexes have been determined. Here, we resolved the pBF2*1501 complex and the cCD8αα/pBF2*1501 and cCD8αα/pBF2*0401 complexes in nonmammals for the first time. Remarkably, cCD8αα/pBF2*1501 and the cCD8αα/pBF2*0401 complex both exhibited two binding modes, including an “antibody-like” mode similar to that of the known mammal CD8/pMHC-I complexes and a “face-to-face” mode that has been observed only in chickens to date. Compared to the “antibody-like” mode, the “face-to-face” binding mode changes the binding orientation of the cCD8αα homodimer to pMHC-I, which might facilitate abundant γδT cells to bind diverse peptides presented by limited BF2 alleles in chicken. Moreover, the forces involving in the interaction of cCD8αα/pBF2*1501 and the cCD8αα/pBF2*0401 are different in this two binding model, which might change the strength of the CD8-pMHC-I interaction, amplifying T cell cross-reactivity in chickens. The coreceptor CD8αα of TCR has evolved two peptide-MHC-I binding patterns in chickens, which might enhance the T cell response to major or emerging pathogens, including chicken-derived pathogens that are relevant to human health, such as high-pathogenicity influenza viruses.

## Introduction

Critical molecules involved in immune defense can be subject to an evolving molecular arms race with all kinds of pathogens, of which the most famous has led to the multiple loci, high allelic polymorphism and high sequence diversity of major histocompatibility complex (MHC) genes (1). These genes encode the classical class I and class II (aka MHC-I/II) molecules that bind antigenic peptides and present them for recognition by the T cell receptor (TCR) on T lymphocytes bearing coreceptors CD8 and CD4, respectively (2). TCR and CD8 cooperatively bind to the peptide-MHC-I complex (aka CD8-pMHC-I), which amplifies the peptide discrimination (3). The CD8-pMHC-I interaction enhances cytotoxic T lymphocyte immunity (4, 5) *via* stabilizing the TCR-pMHC-I interaction (6), recruiting essential signaling molecules to the intracellular side of the TCR-CD3 complex and locating the TCR to specific membrane domains at the cell surface (7–11). Over one million different peptides could be presented by a single classical MHC class I molecule and recognized by a single TCR *via* T cell cross-reactivity, a crucially important phenomenon in immune surveillance (12, 13). In addition, the CD8-pMHC-I interaction extends the range of pMHC-I ligands and is necessary to control the optimal T cell cross-reactivity (14). Indeed, an enhanced level of T cell cross-reactivity in mice was predicted on theoretical grounds (13) because of stronger CD8-pMHC-I interaction than in humans. However, the multiple structural basis of T cell cross-reactivity has now been explained, focusing on the interaction between TCR and pMHC-I and the characteristics of peptide binding (15).

In representative mammals such as humans and mice, only two nonpolymorphic CD8 genes are found, CD8A and CD8B (16). The CD8 dimer exists as a glycoprotein on the T cell surface in two isoforms, CD8αα and CD8αβ. According to current knowledge of human and mouse immunology, CD8α is mainly expressed on the surface of γδT cells, natural killer (NK) cells and dendritic cells (DCs) (17). Despite the different isoforms, CD8αα and CD8αβ show similar affinity for pMHC-I, and both CD8αα and CD8αβ interact with pMHC-I and promote TCR-pMHC-I recognition (18, 19). The chicken is the best-characterized nonmammal model in terms of immunology (20). Both chicken CD8αβ and CD8αα dimers are found on similar cells to those in mammals (21–24). In comparison with the known human and mouse CD8α proteins, chicken CD8α (cCD8α) showed lower diversity in the CDR1-like loop and the CDR2-like loop caused by mutation of their specific amino acid sequences (25, 26). In addition, cCD8αα maintains a few conserved residues with respect to the interaction of human and mouse CD8αα with pMHC-I (26). In contrast to the lower γδ T cell numbers in human and mouse (27, 28), CD8^+^γδT cells represent a major cytotoxic lymphocyte (CTL) subset appearing in the peripheral blood as well as organs such as the gut, spleen, thymus, and bursa of Fabricius of chicken (29–31), constituting up to 50% of peripheral T cells (32, 33). Indeed, after pathogenic bacteria infection, CD8αα^+^ γδ T cell subsets in the spleen, caecum and blood were expanded, further performing CTL immunobiological function (34).

To date, only human and mouse CD8-pMHC-I interactions have been determined; that is, the structures of hCD8αα/HLA-A*0201, hCD8αα/HLA-A*2402, mCD8αα/H-2K^b^ and mCD8αβ/H-2D^d^ have been resolved (19, 35–37). These CD8/pMHC-I structures reveal that CD8 binds to the protruding pMHC-I α3 domain CD loop in an antibody-like manner. Human and mouse CD8 interact with pMHC-I in an allele-dependent but TCR- and peptide-independent manner (38, 39). However, CD8 engagement guides the geometry of TCR-pMHC-I recognition to achieve the intracellular juxtaposition of coreceptor-bound Lck with CD3 ITAMs (34, 40). However, to date, there is still a lack of information on the nonmammalian CD8/pMHC-I complex structure.

In the first comparative analysis, only two residues on the surface of the chicken class I α3 domain were found to be identical with those in mammals, which led to a proposal for the contact site for CD8 binding (41), now amply confirmed by mutagenesis, structural analysis and biophysical analysis in humans and mice (35–37, 42–44). In addition, the chicken class I α3 domain displays moderate levels of allelic polymorphism and sequence diversity (25, 45), suggesting a complementary selective pressure for diversity in the ligand for the polymorphic and polygenic chicken CD8A system. Of further interest is the fact that only the classical class I gene BF2 is expressed at a high level in chickens, and chickens can live or die based on whether pathogen peptides are bound and presented by the dominantly expressed class I molecule (46–48), intensifying the selection for appropriate CD8 binding.

In this paper, we determined amazingly high affinities between chicken CD8αα homodimers and pBF2*1501 compared to mammalian CD8-pMHC-I interactions and derived cocrystals of chicken CD8αα homodimers with pBF2*1501 and pBF2*0401. We first confirmed that CD8 dimers recognize the same CD loop of the α3 domain in two different modes, namely, an “antibody-like” mode and a “face-to-face” mode that does not occur in mammals. The two binding models are very helpful to the understanding of chicken T cell immunity, including the limited MHC-I allelic genes and added specific T cell constituents. In addition, the two binding modes may have developed due to the molecular arms race with major or emerging pathogens, including chicken-derived pathogens that are relevant to human health, such as highly variable and highly pathogenic influenza virus strains.

## Materials and Methods

### Protein Preparation

The peptide RY0808 (RRREQTDY) derived from MDV was synthesized by Invitrogen, USA. The pET21a plasmids encoding chicken CD8α, BF2*1501, BF2*0401, and β2m and another peptide termed IE8 (IDWFDGKE) for prokaryotic expression were maintained in our laboratory (26, 49, 50). Proteins of chicken CD8α (cCD8α), BF2*1501, BF2*0401, and β2m were expressed as inclusion bodies, and the preparation of soluble cCD8α protein was carried out essentially as described previously (26). The cCD8αα protein was purified on a Superdex 200 16/60 column (GE Healthcare, USA). In addition, after the refolding of BF2*1501 and β2m with RY0808 and of BF2*0401 and β2m with the IE8 peptide, the refolded complexes were further purified on a Superdex 200 16/60 column, followed by Resource Q anion-exchange chromatography (GE Healthcare, USA). The purified RY0808-BF2*1501-β2m (pBF2*1501) and IE8-BF2*0401-β2m (pBF2*0401) complexes were buffer-exchanged three times with 10 mM Tris-HCl, 50 mM NaCl, pH 8.0. Next, pBF2*1501, the cCD8αα and pBF2*1501 (cCD8αα/pBF2*1501) complexes and the cCD8αα and pBF2*0401 complexes (cCD8αα/pBF2*0401) were mixed at a 2:1 molar ratio at 277 K overnight. Finally, the chicken protein complexes pBF2*1501, cCD8αα/pBF2*1501, and cCD8αα/pBF2*0401 were diluted to 5 mg ml^-1^ and 10 mg ml^-1^.

### Affinity Analysis by Surface Plasmon Resonance and Superdex75 16/60 Column

Surface plasmon resonance binding experiments were performed on a Biacore3000. pBF2*1501 and cCD8αα protein both were purified with buffer containing 10mM HEPES PH 7.4, 150mM NaCl, 3mM EDTA and 0.005% Tween 20. pBF2*1501 was covalently coupled with CM-5 chip (cat. no. BR-1000-14, Biacore-GE Healthcare, Piscataway, NJ) as the stationary phase. The different concentration of cCD8αα including 0, 0.78, 1.56, 3.125, 6.25, 12.5, 25, and 50 μM were injected as the mobile phase. And then, the data were analyzed with BIA Evaluation Software 3.2. The coupling conditions and data analysis were performed as described previously (51). After the incubation of purified cCD8αα and pBF2*1501 for 3 h at 4°C, the coexistence of chicken cCD8αα and pBF2*1501 complexes on the gel column was tested by using a Superdex 75 16/60 column (GE Healthcare, USA) and sodium dodecyl sulfate-polyacrylamide gel electrophoresis (SDS-PAGE).

### Crystallization and Data Collection

The complexes pBF2*1501, cCD8αα/pBF2*1501 and cCD8αα/pBF2*0401 were screened in crystallization trials by the sitting-drop vapor-diffusion method with the Index, Crystal Screen, Crystal Screen 2, Crystal Screen Cryo, Crystal Screen 2 Cryo, and PEG Ion 1 and 2 Kits (Hampton Research, USA) at 291 K. Crystals from the protein concentration of 10 mg ml^-1^ were observed in PEG Ion Kit No. 16 (0.2 M magnesium nitrate hexahydrate, 20% (*w/v*) polyethylene glycol 3,350, pH 5.8) and Index Kit No. 72 (0.2 M sodium chloride, 25% (w/v) polyethylene glycol 3,350, 0.1 M HEPES pH 7.5) and No. 67 (0.2 M ammonium sulfate, 0.1 M Bis-tris pH 6.5, 25% (w/v) polyethylene glycol 3,350). Diffraction data from chicken pBF2*1501, cCD8αα/pBF2*1501 and cCD8αα/pBF2*0401 complexes were collected on Beamline BL17U at the Shanghai Synchrotron Radiation Facility (SSRF; Shanghai, People’s Republic of China) at a wavelength of 0.97972 Å with an ADSC Q315 CCD detector. The collected diffraction data were indexed and scaled with HKL2000 (52).

### Structure Determination and Refinement

The structures were determined by molecular replacement by using Molrep and Phaser in the CCP4 package, with the structures of chicken CD8αα [Protein Data Bank (PDB) code: 5EB9] and BF2*0401-IE8 [PDB code: 4E0R] as the search models. The construction and refinement of the complex were performed by the programs Coot and Refmac5. Subsequent refinements were conducted for energy minimization, the restriction of individual B factors, and the addition of water molecules, with noncrystallographic symmetry restraints applied to the one molecule of pBF2*1501 and the two molecules of chicken cCD8αα/pBF2*1501 and cCD8αα/pBF2*0401 in the asymmetric unit. Ramachandran plots and secondary structure assignments were generated by SFCHECK (53). The final structures of the complex consisted of pBF2*1501 and two complete chicken cCD8αα/pBF2*1501 and cCD8αα/pBF2*0401 molecules, with R-factor = 0.2304, R-free = 0.2647; R-factor = 0.2533, R-free = 0.2990; and R-factor = 0.2519, R-free = 0.2881, respectively. These crystal structures of cCD8αα/pBF2*1501, cCD8αα/pBF2*0401, and pBF2*1501 have been deposited in the PDB (http://www.pdb.org/pdb/home/home.do) with accession numbers 6LHF, 6LHG and 6LHH.

### Structural Analysis and Generation of Illustrations

The residues involved in the interactions of cCD8αα with pBF2*1501 and pBF2*0401 were identified by the web servers PDBePISA (https://www.ebi.ac.uk/pdbe/pisa/) (13) and Ring (http://protein.bio.unipd.it/ring/) (12). Structural illustrations and electrostatic potential surfaces were generated with the PyMOL molecular graphics system (DeLano Scientific; http://www.pymol.org).

## Results

### An Unexpected Interaction Exists Between cCD8αα and pBF2*1501

**Figure 1:**
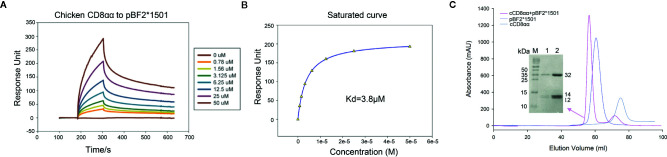
****Affinity measurement by surface plasmon resonance (SPR) and gel column coexistence testing of cCD8αα/pBF2*1501 complex *in vitro*. **(A)** pBF2*1501 was the stationary phase, and cCD8αα diluted to 0, 0.78, 1.56, 3.125, 6.25, 12.5, 25, and 50 μM was the mobile phase. **(B)** The affinity was measured to be Kd=3.8 μM. **(C)** Purification peak map of cCD8αα, pBF2*1501 and cCD8αα/pBF2*1501, which are colored gray, light blue and pink. The peak position of cCD8αα/pBF2*1501 is in front of that of pBF2*1501, and SDS-PAGE identification of the cCD8αα/pBF2*1501 complex peak shows three distinct bands corresponding to BF2*1501, β2m, and cCD8α protein.

**Figure 2:**
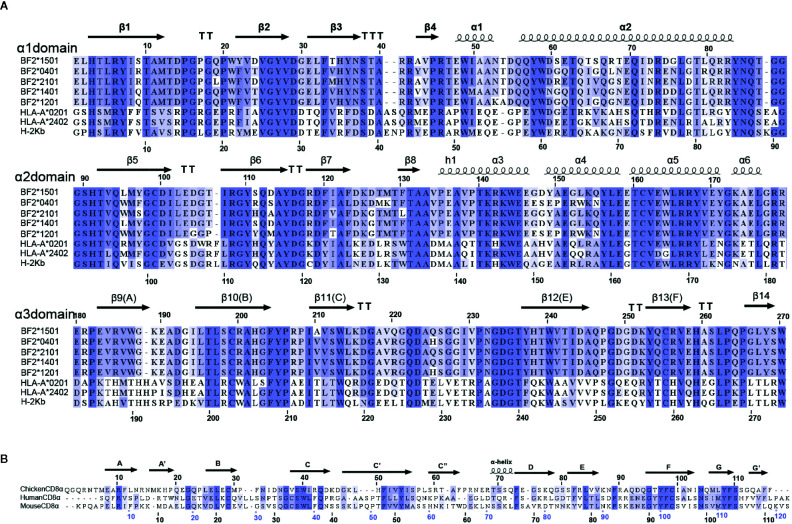
Multiple amino-acid sequence alignments. **(A)** Multiple amino-acid sequence alignment of BF2*1501, BF2*0401, BF2*2101, HLA-A*0201, HLA-A*2402, and H-2K^b^ mature peptides. The α1, α2, and α3 domains of pMHC-I molecules are shown by three lines. The secondary structure and sequence number are marked above the sequence alignment. **(B)** Multiple alignment of chicken, human and mouse CD8α mature peptides. The secondary structure and sequence number are marked above the sequence alignment.

The affinity between cCD8αα and pBF2*1501 (Kd=3.8 μM) is higher than the known binding of CD8 and pMHC-I in mammals (54) (**Figures 1A, B** **)**. Additionally, cCD8αα and pBF2*1501 can stably coexist on a gel column *in vitro* (**Figure 1C**). In addition, the amino acids of chicken BF2 molecules exhibited a large difference from those of humans and mice, especially for the α3 domain, and only 28 conserved residues exist between the α3 domains of BF2 molecules and HLA-A*0201 and mouse H-2K^b^ (**Figure 2A**). In addition, there were only 20 conserved residues between chicken CD8α and human and mouse CD8α (**Figure 2B**). Therefore, an unexpected interaction between chicken CD8α and pMHC-I was distinct from those in humans and mice.

To investigate the interaction of CD8 and classical class I molecules in chickens, cocrystals of pBF2*1501 with peptide RRREQTDY (RY0808), of cCD8αα homodimers with the pBF2*0401 molecule and the peptide IDWFDGKE (IE8) were formed as previously reported (49), as well as cCD8αα homodimers with the pBF2*1501 molecule just described, which diffracted to 2.7 Å, 2.6 Å, and 2.8 Å and belonged to the P3_1_2_1,_ P1, and P2_1_ space groups, respectively (**Table 1**).

**Table 1:** X-ray diffraction data processing and refinement statistics.

Statistic Value for:	cCD8αα/pBF2*0401	cCD8αα/pBF2*1501	BF2*1501/RY0808
Data processing			
Space group	P2_1_	P1	P3_1_2_1_
Cell parameters			
a (Å)	90.576	51.34	123.45
b (Å)	90.82	66.73	123.45
c (Å)	94.87	103.95	81.22
α (°)	90.00	84.60	90.00
β (°)	98.61	82.04	90.00
γ (°)	90.00	67.51	120.00
Resolution range (Å)	50.00-2.80(2. 90–2.80)	50.00-2.6(2.69–2.6)	50.00-2.71(2.77–2.71)
Total reflections	163307	108500	111237
Unique reflections	37565	36586	18044
Completeness (%)	99.57 (97.75)	95.34 (93.09)	96.2(97.7)
Redundancy	4.3(4.5)	3.0(3.1)	3.4(2.1)
*R*merge (%)	10.5(61.3)	10.9(51.6)	8.0(24.00)
I/σ (I)	10.21 (3.04)	8.25 (3.24)	11.09(2.659)
Refinement			
*R*work	0.2519	0.2533	0.2304
*R*free	0.2881	0.2990	0.2647
RMSD			
Bond lengths (Å)	0.005	0.010	0.004
Bond angles (°)	1.16	1.68	0.865
Average B factor	80.60	58.40	59.58
Ramachandran plot			
Most favored (%)	97	96	97.1
Disallowed (%)	0.17	0	0

### cCD8αα Binding Shows a “Pull” of the CD Loop of pMHC-I

**Figure 3:**
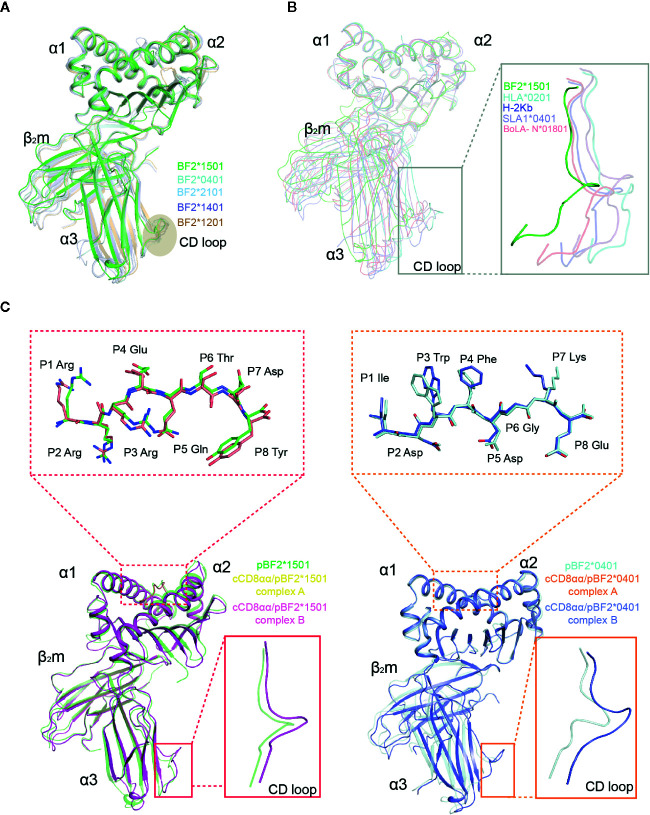
Superposition of pBF2*1501 with other known chicken pMHC-I structures, representative mammal pMHC-I structures and cCD8αα/pBF2*1501 and cCD8αα/pBF2*0401 complexes. **(A)** Superimposed Cα traces of chicken BF2*1501-RY0808-β2m (green), pBF2*0401 (PDB code: 4E0R, cyan), pBF2*2101 (PDB code: 3BEV, light blue), pBF2*1401 (PDB code: 4CW1, purple), and pBF2*1201 (PDB code: 5YMV, brown). **(B)** Cα-trace comparison of BF2*1501-RY0808-β2m (green), HLA-A*0201 (PDB code: 1HHK, cyan), H-2K^b^ (PDB code: 1G7Q, blue), SLA-1*0401 (PDB code: 3QQ3, light purple), and BoLA-N*01801 (PDB code: 3PWU, light red), with an enlarged view of the CD loop in the gray box. **(C)** Overlap between free pBF2*1501 and pBF2*1501 of cCD8αα/pBF2*1501 and between free pBF2*0401 and pBF2*0401 of cCD8αα/pBF2*0401, based on the α1 and α2 domains, respectively. Enlarged views of the overlapped peptides are shown in the dotted red box and dotted orange box for cCD8αα/pBF2*1501 and cCD8αα/pBF2*0401, respectively; an enlarged view of the overlapped α3 domain CD loop is shown in the red box and orange box for cCD8αα/pBF2*1501 and cCD8αα/pBF2*0401. Free pBF2*1501, pBF2*1501 of cCD8αα/pBF2*1501 complex A, pBF2*1501 of cCD8αα/pBF2*1501 complex B, free pBF2*0401, pBF2*0401 of cCD8αα/pBF2*0401 complex A and pBF2*0401 of cCD8αα/pBF2*0401 complex B are colored green, yellow, light pink, cyan, orange, and light blue, respectively.

The BF2*1501 complex consists of α1, α2, α3 domains of the heavy chain, a light chain cβ2m, and the 8-mer peptide RRREQTDY (henceforth called RY0808), which is extremely similar to other known chicken class I molecules including BF2*2101 (3BEV), BF2*0401 (40ER), BF2*1401 (4CW1), BF2*1201 (5YMV) with Cα root mean square deviations (RMSDs) value of 0.83 Å, 0.54 Å, 0.51 Å and 0.82 Å respectively; the complexes also share the closely similar α3 domain CD loop (**Figure 3A**). However, the chicken BF2 structures differ more from human, mouse, bovine, and swine MHC class I structures, with Cα RMSD values of 2.61 Å, 2.11 Å, 1.98 Å, and 1.91 Å, especially for the α3 domain CD loops, which are shifted away from the human and mouse CD8 orientation by approximately 3.4-3.7 Å, which theoretically should result in a spatial relationship between cCD8 and the α3 domain of pMHC-I in chickens (**Figure 3B**). This different conformation of the α3 domain CD loop might lead to the distinct binding mode between CD8 and pMHC-I complexes in chickens and mammals because of the narrow space for CD8 engagement in chickens.

**Figure 4:**
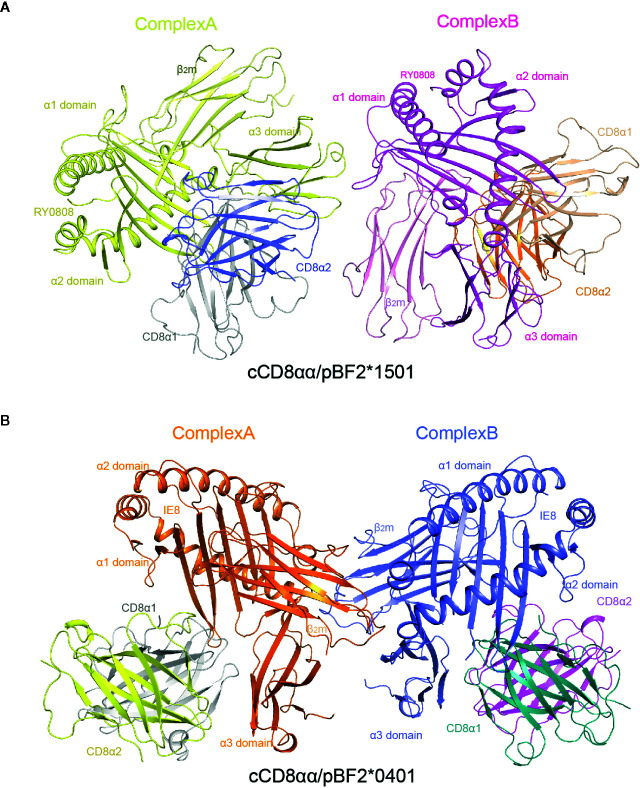
Overall view of chicken cCD8αα/pBF2*1501 and cCD8αα/pBF2*0401 complexes. **(A)** An asymmetric unit contains two chicken cCD8αα/pBF2*1501 complexes, namely, complex A and complex B, each consisting of pBF2*1501 (BF2*1501, chicken β2m, and the peptide RY0808) and the cCD8αα homodimer. pBF2*1501, the cCD8α1 subunit, and the cCD8α2 subunit of complex A are colored yellow, gray and blue, respectively. pBF2*1501, the cCD8α1 subunit, and the cCD8α2 subunit of complex B are colored pink, light brown and orange, respectively. **(B)** An asymmetric unit contains two chicken cCD8αα/pBF2*0401 complexes, namely, complex A and complex B, each consisting of pBF2*0401 (BF2*0401, chicken β2m, and the peptide IE8) and the cCD8αα homodimer. pBF2*0401, the cCD8α1 subunit, and the cCD8α2 subunit of complex A are colored orange, gray and yellow, respectively. pBF2*0401, the cCD8α1 subunit, and the cCD8α2 subunit of complex B are colored blue, deep green, and pink, respectively.

Similar to the human hCD8αα/pHLA-A*2402 and mouse mCD8αα/pH-2K^b^ complexes, each unit cell of cCD8αα/pBF2*1501 and cCD8αα/pBF2*0401 contained two asymmetric complexes, each with one class I heterotrimer (RY0808/BF2*1501/β2m and IE8/BF2*0401/β2m) and one CD8αα homodimer, termed complex A and complex B (**Figure 4**). In addition, cCD8αα binding did not change the main chain of the peptides, except that some side chains of the nonanchoring peptide residues became flexible in both the cCD8αα/pBF2*0401 and cCD8αα/pBF2*1501 complexes, which did not alter the peptide binding to BF2*1501, BF2*0401 and the corresponding TCR (**Figures 3C** and **S1A**). However, compared to the pBF2*1501 complex, the α3 domain CD loop of complex A and complex B with intact mesh were pulled towards cCD8αα by approximately 4.2 Å and 3.8 Å in the cCD8αα/pBF2*1501 complex. Complexes A and B of cCD8αα/pBF2*0401 also moved towards cCD8αα by approximately 4.3 Å and 4.0 Å (**Figures 3C** and **S1B**). Moreover, the crucial protruding α3 domain CD loop differs in direction, and the protruding loop (220~228) in the CD loop of the α3 domain is an important region for CD8 binding independent of species (43, 55, 56) (**Figure 3C**). This phenomenon is completely different from the “pull” and “push” binding of the MHC-I α3 domain CD loop adopted by complexes A and B, respectively, in humans and mice (35, 36).

### Chicken cCD8αα/pMHC-I Complexes Demonstrate Two Different cCD8αα Engagement Modes

**Figure 5:**
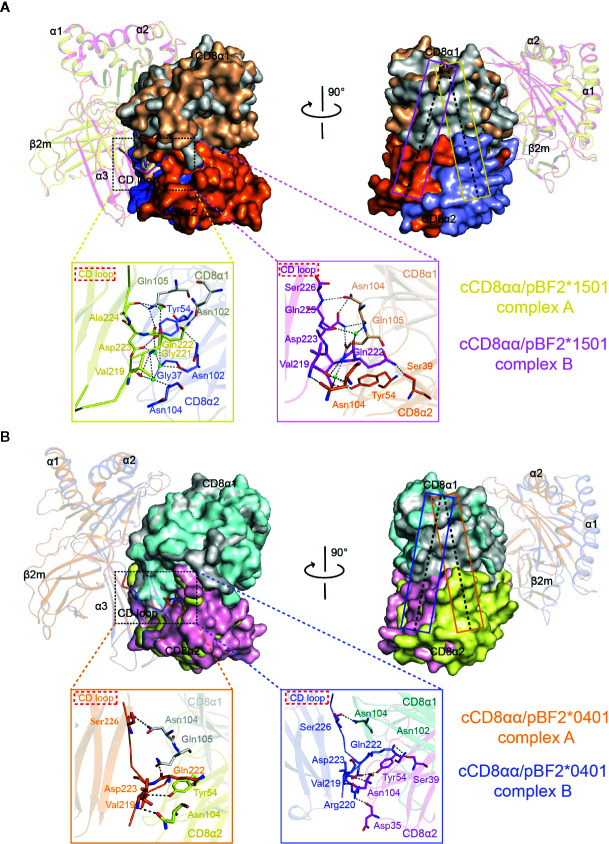
Superposition of complex A and complex B of cCD8αα/pBF2*1501 and cCD8αα/pBF2*0401 using the Cα atoms of domains α1 and α2. **(A)** Overlap of complex A and complex B of cCD8αα/pBF2*1501 based on the Cα atoms of domains α1 and α2, as shown from the front view and in a clockwise rotation by 90°. The interaction details between the α3 domain CD loop and the cCD8αα homodimer are shown in an enlarged view in yellow and pink boxes for complex A and complex B, respectively. In complex A, the contact residues are shown in stick representation, and the residues of BF2*1501 are colored yellow. The residues of cCD8α1 are colored gray, the residues of cCD8α2 are colored light blue, and the interaction forces are marked by black dotted lines. In complex B, the contact residues are shown in stick representation, and the residues of BF2*1501 are colored light pink. The residues of cCD8α1 are colored light brown, the residues of cCD8α2 are colored orange, and the interaction forces are marked by black dotted lines. pBF2*1501 of cCD8αα/pBF2*1501 complex A and cCD8αα/pBF2*1501 complex B are colored yellow and light pink; the cCD8α1 and cCD8α2 subunits of the cCD8αα/pBF2*1501 complex A are colored light gray and light blue; and the cCD8α1 and cCD8α2 subunits of cCD8αα/pBF2*1501 complex B are colored light brown and orange. The yellow and pink rectangles represent the central plane of the cCD8αα homodimer of complex A and complex B of cCD8αα/pBF2*1501, respectively. **(B)** Overlap of complex A and complex B of cCD8αα/pBF2*0401 based on the Cα atoms of domains α1 and α2, shown from the front view and rotated clockwise by 90°. The interaction details between the α3 domain CD loop and the cCD8αα homodimer are shown in enlarged views in light orange and light blue boxes for complex A and complex B, respectively. In complex A, the contact residues are shown in stick representation, and the residues of BF2*0401 are colored light orange, the residues of cCD8α2 are colored yellow, and the interaction forces are marked by black dotted lines. In complex B, the contact residues are shown in stick representation, the residues of BF2*0401 are colored light blue, the residues of cCD8α1 are colored deep green, the residues of cCD8α2 are colored pink, and the interaction forces are marked by black dotted lines. pBF2*0401 of cCD8αα/pBF2*0401 complex A and cCD8αα/pBF2*0401 complex B are colored light orange and light blue; the cCD8α1 and cCD8α2 subunits of cCD8αα/pBF2*0401 complex A are colored gray and yellow; and the cCD8α1 and cCD8α2 subunits of cCD8αα/pBF2*0401 complex B are colored deep-green and pink. The orange and blue rectangles represent the central plane of the cCD8αα homodimer of complex A and complex B of cCD8αα/pBF2*0401, respectively.

The CD loop of complex B lies closer to the cCD8αα homodimer, and the whole cCD8αα homodimer clearly skews towards the α2 domain and β2m in complex A compared to complex B (**Figure 5**). The hCD8αα/pHLA-A*0201, hCD8αα/pHLA-A*2402 and mCD8αα/pH-2K^b^ complexes proved that the CD loop of α3 domain forms hydrogen bonds and salt bridges with CD8αα. The α3 domain CD loop is clamped by all six CDR-like loops of CD8αα, among which Gln226 is the most important residue and is conserved across different species.

In cCD8αα/pBF2*1501 complex A, the corresponding residue Gln222 only forms two hydrogen bonds with Asn102 on F strand and Gln105 on CDR3-like loop of the cCD8α1 subunit and forms one hydrogen bond with Asn102 on F strand of the cCD8α2 subunit instead of the conserved C strand that located on the deep of CD8 cavity in humans and mice. Moreover, Ala224 of α3 domain CD loop forms one hydrogen bond with Gln105 of the cCD8α1 CDR3-like loop, and Asp223 of α3 domain CD loop forms one hydrogen bond with Tyr54 of the cCD8α2 C′ strand in complex A (**Figure 5A**). Besides that, four water molecules participate in the interactions and forms a forces network between α3 domain CD loop and cCD8α subunits (**Figure 5A**). In cCD8αα/pBF2*0401 complex A, the corresponding residue Gln222 only forms one hydrogen bond with Gln105 on CDR3-like loop of the cCD8α1 subunit. Another two hydrogen bonds are formed between Val219 and Ser226 of α3 domain CD loop and Asn104 of the cCD8αα CDR3-like loop, and Asp223 of α3 domain CD loop forms one hydrogen bond with Tyr54 of the cCD8α2 C′ strand in complex A (**Figure 5B**). Therefore, Gln222 of BF2*1501 and BF2*0401 α3 domain CD loops is not a significant residue, which inserts into the deep of CD8 cavity and contribute to the antibody-like interaction model existing in the human and mouse CD8/pMHC-I complex. The corresponding residue Gln226 in the mammalian CD8αα/pMHC-I complex is the most important residue contributing to the antibody-like binding model of the mammalian CD8αα/pMHC-I complex (35–37). The special interaction contributed by important residue Gln222 and other distinct interactions between cCD8αα and the CD loop of the α3 domain leads to the rotation of complex A towards the α2 domain and β2m in comparison to complex B, which finally creates a novel binding mode that we termed the “face-to-face” mode (**Figure 5** and **Table 2**).

**Table 2:** Statistics of forces in cCD8αα/pBF2*1501 and cCD8αα/pBF2*0401 complexes.

Forces	cCD8αα/BF2*1501complex A	cCD8αα/BF2*1501complex B	cCD8αα/BF2*0401 complex A	cCD8αα/BF2*0401 complex B
**Hydrogen bonds (H)**	7 H	12 H	8 H	11 H
**Salt bridges (S)**	1 S	1 S	2 S	1 S
**Van der Waals (vdw)**	27 vdw	34 vdw	11 vdw	14 vdw

However, cCD8αα/pBF2*1501 complex B and cCD8αα/pBF2*0401 complex B present a parallel binding model of cCD8αα interaction with hCD8αα/pHLA-A*0201 and mCD8αα/pH-2K^b^. In the CD loop of the pBF2*1501 α3 domain, Ser226 forms one hydrogen bond with Asn104 of the CDR3-like loop of cCD8α1 subunit; Gln225 forms two hydrogen bonds with Asn104 of the CDR3-like loop of cCD8α1 subunit; Asp223 forms one hydrogen bond with Tyr54 of the cCD8α2 C′ strand; Val219 forms two hydrogen bonds with Asn104 of the cCD8α2 CDR3-like loop, and the important residue Gln222 forms one hydrogen bond with Ser39 of the cCD8α2 C strand and a water molecule participates in the stabilization of Gln222 (**Figure 5A**). In the pBF2*0401 α3 domain CD loop, in addition to two hydrogen bonds formed by Val219 with Asn104 of cCD8α2, the Arg220 forms one hydrogen bond with Asp35 of cCD8α2 CDR1-like loop, the Asp223 forms one hydrogen bond with Tyr54 of the cCD8α2 C′ strand, the Ser226 forms one hydrogen bond with Asn104 of the cCD8α1 CDR3-like loop, and the protruding residue of Gln222 forms two hydrogen bonds with Ser39 of the cCD8α2 C strand (**Figure 5B**). Thus, the two cCD8α subunits clamp the CD loop, and the key residue Gln222 inserts into the deep of CD8 cavity and plays vital roles in the interactions of complexes B, which is similar to the classical binding mode in mammalian CD8/pMHC-I complexes.

### The “Face-to-Face” Mode Causes Different and Fewer Interactions in Complex A

**Figure 6:**
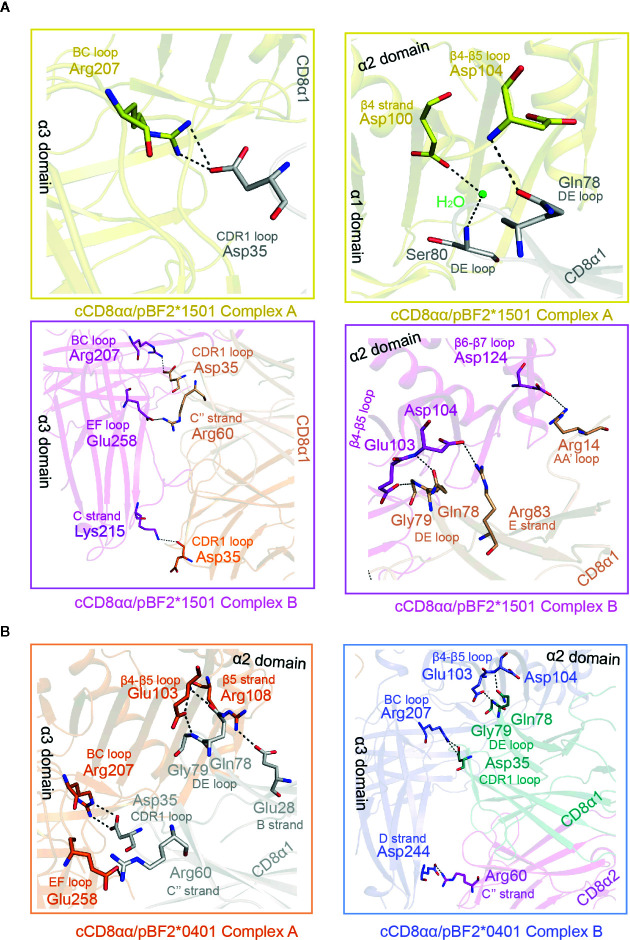
Details of the interaction between the cCD8αα homodimer and pBF2*1501 and pBF2*0401 of complex A and complex B. **(A)** The interaction between cCD8αα homodimer and the α1, α2, and α3 domains (exclusive of the CD loop) of pBF2*1501. The interactions of complex A are shown in enlarged views in yellow boxes. The contact residues are marked by sticks and labeled by three-letter abbreviation and sequence number, and the interaction forces are marked by black dotted lines. In complex A, the contact residues of pBF2*1501 are colored yellow, the contact residues of the cCD8α1 subunit are colored gray, and the contact residues of the cCD8α2 subunit are colored blue. The interactions of complex B are shown in an enlarged view in a pink box. The contact residues are shown in stick representation and labeled by three-letter abbreviation and sequence number, and the interaction forces are marked by the black dotted line. In complex A, the contact residues of pBF2*1501 are colored pink, and the contact residues of the cCD8α1 subunit are colored light brown. **(B)** The interaction between the cCD8αα homodimer and the α1, α2, and α3 domains (exclusive of the CD loop) of pBF2*0401. The interactions of complex A are shown in an enlarged view in an orange box. The contact residues are shown in stick representation and labeled by three-letter abbreviation and sequence number, and the interaction forces are marked by black dotted lines. In complex A, the contact residues of pBF2*0401 are colored orange, and the contact residues of the cCD8α1 subunit are colored gray. The interactions of complex B are shown in an enlarged view in a pink box. The contact residues are shown in stick representation and labeled by three-letter abbreviation and sequence number, and the interaction forces are marked by black dotted lines. The contact residues of pBF2*1501 are colored blue, the contact residues of the cCD8α1 subunit are colored deep green, and the contact residues of the cCD8α2 subunit are colored pink.

In addition to those in the CD loop, other differences exist in the α2, β2m and α3 domains of cCD8αα/pBF2*1501 and cCD8αα/pBF2*0401 complexes A and B (**Figure 6** and **Supplementary Table 1**). In cCD8αα/pBF2*1501 complex A, Arg207 of the α3 domain BC loop forms one hydrogen bond and one salt bridge with Asp35 of the cCD8α1 CDR-like loop (**Figure 6A**). However, in cCD8αα/pBF2*1501 complex B, one hydrogen bond was formed between Arg207 and Asp35, and Glu258 forms one salt bridge with Arg60 of cCD8α1, Lys215 forms one hydrogen bond with Asp35 of cCD8α2 (**Figure 6A**). In the cCD8αα/pBF2*0401 complex A, the corresponding residue Arg207 of the α3 domain BC loop forms one hydrogen bond and one salt bridge with Asp35 of the cCD8α1 CDR1-like loop, and the corresponding residue Glu258 of the α3 domain E strand forms one salt bridge with Arg60 of the cCD8α1 C′′ strand (**Figure 6B**). In contrast, the corresponding residues Arg207 forms two hydrogen bonds with Asp35 of cCD8α1, and Glu244 forms one salt bridge with Arg60 of the cCD8α2 C′′ strand in cCD8αα/pBF2*0401 complex B (**Figure 6B**).

In the α2 domain of cCD8αα/pBF2*1501 complex A, Asp104 of the β4-β5 loop forms one hydrogen bond with Gln78 of the cCD8α1 DE loop, and Asp100 of the β4 strand contacts to Ser80 of the cCD8α1 DE loop with the help of water molecule (**Figure 6A**). However, in cCD8αα/pBF2*1501 complex B, Glu103 and Asp104 of the β4-β5 loop form two hydrogen bonds with Gly79 and Gln78 of the cCD8α1 DE loop, and Asp124 of the β6-β7 loop forms one hydrogen bond with Arg14 of the cCD8α1 AB loop (**Figure 6A**). In cCD8αα/pBF2*0401 complex A, the residue Arg108 of the β5 strand forms one hydrogen bond with Glu28 of the CD8α1 subunit B strand, Glu103 of the β4-β5 loop forms two hydrogen bonds with Gln78 and Gly79 of the cCD8α1 DE loop (**Figure 6B**). In contrast, the residue Glu103 and Asp104 of the β4-β5 loop form one hydrogen bond with Gly79 and Gln78 of the cCD8α1 subunit DE loop respectively, in cCD8αα/pBF2*0401 complex B (**Figure 6B**). In addition to hydrogen bonds and salt bridges, six van der Waals contacts are contributed by residues of BF2*1501 except for α3 CD loop and eleven van der Waals contacts are contributed by β2m to the interaction in cCD8αα/pBF2*1501 complex A (**Supplementary Table 1**). In cCD8αα/pBF2*1501 complex B, twelve van der Waals contacts formed by residues of BF2*1501 except for α3 CD loop and only five van der Waals contacts are contributed by β2m (**Supplementary Table 1**). Besides forces contributed by the α3 CD loop, other stable interactions, including hydrogen bonds, salt bridges and van der Waals interactions, that assist in drawing the pBF2*1501 and pBF2*0401 molecules close to cCD8αα are also mostly different in complex A and complex B. Therefore, the affinity should be different between cCD8αα and pBF2*1501 and pBF2*0401 in complex A and complex B. In both cases, complex B might possess a higher affinity between cCD8αα and pBF2*1501 and pBF2*0401 than that of complex A, because more forces including hydrogen bonds and van der Waals interactions predominate between cCD8αα and pBF2*1501 and pBF2*0401 in complex B (**Table 2**). However, the solvents might influence the affinity between cCD8αα and pBF2*1501 in complex A.

### Specific Interaction Features Between cCD8 and pMHC-I Outside of Mammals

**Figure 7:**
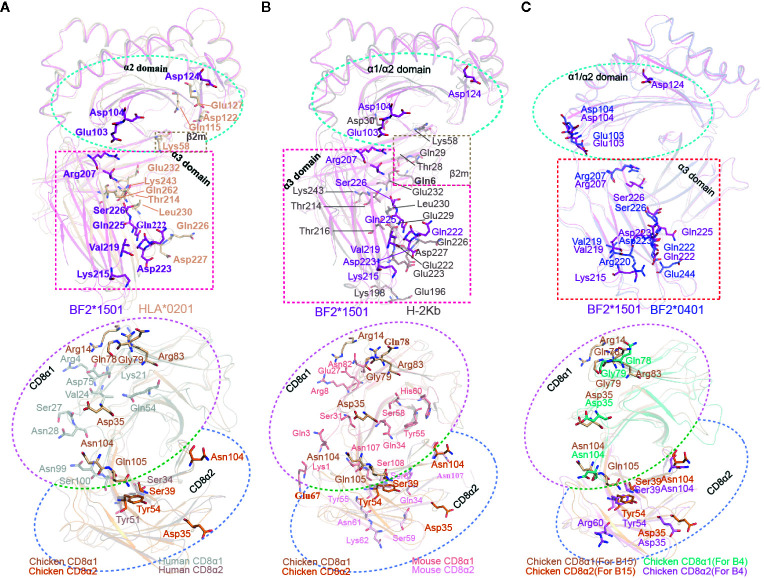
Contact residue conservation analysis among cCD8αα/pBF2*1501 complex B, cCD8αα/pBF2*0401 complex B, hCD8αα/pHLA-A*0201, mCD8αα/pH-2K^b^ complexes. **(A)** Conservation analysis of contact residues between cCD8αα/pBF2*1501 complex B and the hCD8αα/pHLA-A*0201 complex. The alignment between pBF2*1501 and pHLA-A*0201 is shown at the top, and the alignment between cCD8αα and hCD8αα is shown below. All contact residues are shown in stick representation and labeled by three-letter abbreviation. The contact residues of pBF2*1501 and pHLA-A*0201 are colored pink and light brown, and the cyan rectangle represents the α2 domain, while the pink rectangle represents the α3 domain. The contact residues of the cCD8α1 and cCD8α2 subunits are colored light brown and orange, and the contact residues of the hCD8α1 subunit and hCD8α2 subunit are colored gray and brown. The light pink circle represents the CD8α1 subunit, and the light-blue circle represents the CD8α2 subunit. **(B)** Conservation analysis of contact residues between cCD8αα/pBF2*1501 complex B and the mCD8αα/pH-2K^b^ complex. The alignment between pBF2*1501 and pH-2K^b^ is shown at the top, and the alignment between cCD8αα and mCD8αα is shown below. All contact residues are shown in stick representation and labeled by three-letter abbreviation. The contact residues of pBF2*1501 and pH-2K^b^ are colored pink and dark gray, and the cyan rectangle represents the α2 domain, while the pink rectangle represents the α3 domain. The contact residues of the cCD8α1 subunit and cCD8α2 subunit are colored light brown and orange, and the contact residues of the mCD8α1 subunit and mCD8α2 subunit are colored light red and light pink. The light-pink circle represents the CD8α1 subunit, and the light-blue circle represents the CD8α2 subunit. **(C)** Conservation analysis of contact residues between cCD8αα/pBF2*1501 complex B and cCD8αα/pBF2*0401 complex B. The alignment between pBF2*1501 and pBF2*0401 is shown at the top, and the alignment of cCD8αα is shown below. All contact residues are shown in stick representation and labeled by three-letter abbreviation. The contact residues of pBF2*1501 and pBF2*0401 are colored pink and blue, and the cyan rectangle represents the α2 domain, while the pink rectangle represents the α3 domain. The contact residues of the cCD8α1 subunit and the cCD8α2 subunit of cCD8αα/pBF2*1501 complex B are colored light brown and orange, and the contact residues of the cCD8α1 and cCD8α2 subunits of cCD8αα/pBF2*0401 complex B are colored deep green and pink. The light-pink circle represents the CD8α1 subunit, and the light-blue circle represents the CD8α2 subunit.

Complex B of cCD8αα/pBF2*1501 and complex B of cCD8αα/pBF2*0401 share a similar CD8 interaction mode with mammalian MHC-I, but the amino acids of BF2*1501 and BF2*0401 especially for the α3 domain is different from human and mouse pMHC-I molecules (**Figure 2**). Moreover, the amino acid identity of CD8α is low in chicken, human and mouse (**Figure 2**). These results reveal that chicken cCD8αα-pMHC-I interaction represents a special feature differing from humans and mice. Corresponding to sequence homology, the interface forces among chicken, human and mouse CD8αα/pMHC-I showed substantial differences (**Figure 7**). Only two residues, Gln222 and Asp223 of BF2*1501, corresponding to Gln226 and Asp227 in human and mouse MHC-I, and three residues that participate in the complex interaction, Ser39, Tyr54 and Asn104 of cCD8α, are conserved between chicken and mammals (**Figures 7A, B** **)**.

In the case of hCD8αα/pHLA-A*0201, mutagenesis of any of the three residues Gln115, Asp122 and Glu128 in the HLA-A*0201 α2 domain can abolish hCD8αα binding (37). However, three different residues, Glu103, Asp104 and Asp124, in the BF2*1501 α2 domain form contacts with cCD8αα (**Figure 7A**). Moreover, mutagenesis data on the α3 domain of the human and mouse complexes showed that three clusters of α3 domain residues (residues 220–232, 233–235, and 245–247) and especially the MHC-I α3 CD loop (residues 220–228) are key to the CD8αα-pMHC-I interaction (43, 56). However, in chickens, in addition to Gln222 and Asp223, another five nonconserved residues of BF2*1501, Arg207, Lys215, Val219, Gln225, Ser226, and Glu258, are the main contributors to the interaction with cCD8αα (**Figures 7A, B** **)**. Among the seven residues, five of them fall into the cluster 220-228, but two residues, Val219 and Gln225, are specific to chickens. Furthermore, Arg207 is located on the BC loop of the BF2*1501 α3 domain, which is absent in the interactions between CD8α and HLA-A*0201 and H-2K^b^, and no residues of the BC loop participate in the interaction in humans and mice. Another three residues, Lys215, Ser226, and Glu258, are also species specific, although the corresponding residues Glu229 and Gln262 contribute to the interactions in mCD8αα/pH-2K^b^ and hCD8αα/pHLA-A*0201, respectively.

Like the BF2*1501 molecules, most of the contact residues in cCD8αα are chicken specific. For the cCD8α1 subunit, a total of seven residues, namely, Gln78, Gly79, and Arg83 of the DE loop, Arg14 of the AA’ loop, Asp35 of the CDR1 loop, Asn104, Gln105 of the CDR3 loop, contribute to the binding to BF2*1501 (**Figure 7A**). In addition to Asn104, six other residues are nonconserved among chicken and human and mouse, and no corresponding residues form contacts in hCD8α1 and mCD8α1 (**Figures 7A** and **S1B**). However, three of four contact residues in the CD8α2 subunit are conserved among cCD8α and hCD8α and mCD8α, including Ser39, Tyr54 and Asn104, because residues Ser39 and Tyr54 form contacts with Gln222, and these interactions are conserved between chicken complex B and the mammalian CD8/pMHC-I complex (**Figures 7A, B** **)**. Another residue, Asp35, of the CDR1 loop is completely species specific in chickens (**Figures 7C** and **2B**).

## Discussion

The immune molecules of birds, represented by chickens, have low amino acid homology to the corresponding human molecules, but the topological structures of their protein complexes are similar. However, in essence, the 3D structures of chicken immune molecules have their own species characteristics, which leads to some differences in immunobiology. The current study determined the structures of chicken CD8/pMHC-I complexes for the first time, namely, cCD8αα/pBF2*1501 and cCD8αα/pBF2*0401, which each contain complex A and complex B in an asymmetrical unit. cCD8αα binds to pBF2*1501 and pBF2*0401 in an allele-dependent but peptide-independent manner, as demonstrated by the CD8αα-pMHC-I interaction in previous studies (38, 39). Remarkably, cCD8αα binds to pBF2*1501 and pBF2*0401 in complex A of cCD8αα/pBF2*1501 and cCD8αα/pBF2*0401 in a novel “face-to-face” mode, which is distinct from the antibody-like binding mode of the known human and mouse CD8/pMHC-I complexes (19, 35–37). The results showed the heterogeneity of the CTL population and the diversity of its binding with pMHC-I.

Complex B shares the same antibody-like binding mode with the known CD8/pMHC-I complexes in mammals, of which the protruding CD loop was clamped by the CDR loops of cCD8α. It has been proven that CD8 and TCR cooperatively bind pMHC-I and enhance peptide discrimination (57). The stalk regions of CD8 are interpreted to be highly flexible, but O-glycosylation may significantly restrict the flexibility of the stalks and the mobility of the CD8 head group relative to the T cell membrane (58–60). Therefore, “face-to-face” binding causes the cCD8αα binding orientation to pBF2*1501 and pBF2*0401 to skew towards the α2 domain and β2m, which might facilitate larger numbers of γδ TCR to bind diverse peptides presented by limited BF2 alleles in chicken. No structures of chicken TCR-pMHC-I or TCR-pMHC-I-CD8 are yet available in chickens, but the geometry of the TCR-pMHC-I-CD8 complex could modulate TCR signaling and thereby directly impact T cell development and T cell activity.

Moreover, the “face-to-face” binding mode leads to the loss of most hydrogen bonds and salt bridges between the CD loop of BF2*1501 and BF2*0401 and cCD8αα. However, more water molecules contribute to the interaction between the CD loop of BF2*1501 and BF2*0401 and cCD8αα in complex A. Importantly, the MHC-I α3 CD loop (residues 220-228) has been proven to be key for the CD8αα-pMHC-I interaction (43, 56). So, these distinct interactions between the CD loop and cCD8αα might result in the different affinity of CD8-pMHC-I interaction in two binding model. Several studies have demonstrated that CD8 can affect the TCR-pMHC-I interaction that determines the consequences of Ag engagement (61–66). Increasing the strength of the CD8-pMHC-I interaction could substantially increase the number of recognized peptides (67). The CD8-pMHC-I interaction strength can optimize the degree of cross-reactivity and Ag sensitivity of CD8 T cells at various stages of their development, and this coreceptor recognition system has coevolved to provide an unparalleled solution to the unique challenges of effective T cell immunity and is necessary to regulate the balance between optimal cross-reactivity and cognate Ag specificity (14). Moreover, it has been reported that O-glycan sialylation of CD8 modulates the affinity for pMHC-I complex binding with little or no effect on the overall structure of CD8 (59, 68). Thus, the antibody-like binding and “face-to-face” binding modes coexist in equilibrium, which might be a “clever” and important strategy that plays an important role in Ag recognition and T cell cross-reactivity during peripheral antigen recognition.

In conclusion, the coexistence of two binding modes in chicken CD8/pMHC-I complexes would be a result of the molecular arms race between pathogens and chickens, which might enhance the T cell response to major or emerging pathogens, including chicken-derived pathogens that are relevant to human health. This phenomenon might be relevant to the special chicken CTL immune response, especially for the high proportion of approximately 50% of CD8^+^γδT cells in peripheral T cells. It is worth emphasizing that chicken CD8αα binding pMHC-I in complex A also provides a new reference model for human T cell therapy.

## Data Availability Statement

The coordinates and structural characteristics of pBF2*1501, cCD8αα/pBF2*1501, cCD8αα/pBF2*0401 have been deposited in the Protein Data Bank under accession numbers 6LHF, 6LHG and 6LHH.

## Author Contributions

YL crystalized the pBF2*1501, cCD8αα/pBF2*1501, and cCD8αα/pBF2*0401 and solved the structures with the help of RC, RL, LZ, BS, and YW. All the research processes were conducted under the supervision of CX. The draft of manuscript was written by YL and revised by JK and CX. All authors contributed to the article and approved the submitted version.

## Funding

This work was supported by National Natural Science Foundation of China (grant nos.: 31572493 and 31972683).

## Conflict of Interest

The authors declare that the research was conducted in the absence of any commercial or financial relationships that could be construed as a potential conflict of interest.
