# Supplementary material for: The Combination of CD8αα and Peptide-MHC-I in a Face-to-Face Mode Promotes Chicken γδT Cells Response
Source: Front Immunol. 2020 Nov 23;11:605085. doi: 10.3389/fimmu.2020.605085 (PMC7719794; doi:10.3389/fimmu.2020.605085)
Supplement: Supplementary file 1 [file DataSheet_1.docx]

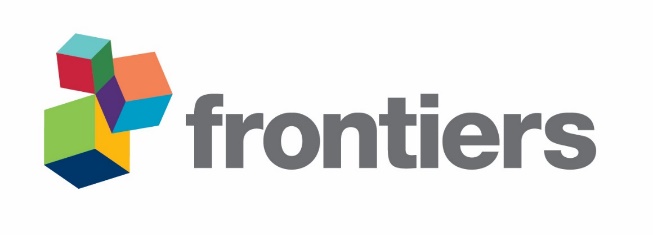


Supplementary Material

# Supplementary Table

**Supplementary Table 1**. **Statistics of Van der Waals in cCD8αα/pBF2*1501 and cCD8αα/pBF2*0401 complexes**

| **cCD8αα/pBF2*1501**  **complex A** | | **cCD8αα/pBF2*1501**  **complex B** | | **cCD8αα/pBF2*0401 complex A** | | **cCD8αα/pBF2*0401 complex B** | |
| --- | --- | --- | --- | --- | --- | --- | --- |
| **BF2*1501** | **cCD8α1** | **BF2*1501** | **cCD8α1** | **BF2*0401** | **cCD8α1** | **BF2*0401** | **cCD8α1** |
| Leu102 | Gln78 | Leu102 | Gln78 | Pro208 | Leu58 | Lys125 | Arg14 |
| Pro208 | Leu58 | Pro208 ^2^ | Leu58 | Ile209 | Leu58 | Pro208 | Leu58 |
| Gln222 ^3^ | Asn102 | Ile209 | Leu58 | Val210 ^2^ | Pro57,Leu58 | Ile209 | Leu58 |
| Ala224 | Gln105 | Gln222 ^3^ | Asn102,  Gln105 | Gln222 | Asn102 | Val210 ^2^ | Leu58, Pro57 |
| Ile229 ^2^ | Asp35 | Gln225 ^3^ | Gln105 | His225 | Gln105 | Gln222 ^3^ | Asn102, Gln105, Leu107 |
| Glu258 ^2^ | Leu58, Arg60 | Ile229 ^2^ | Asp35 |  |  | His225 | Gln105 |
|  |  | Glu258 ^2^ | Leu58 |  |  |  |  |
| **β2m** | **cCD8α1** | **β2m** | **cCD8α1** |  |  |  |  |
| Asp57 ^4^ | Phe32 | Asp58 ^5^ | Phe32 |  |  |  |  |
| Asp58 ^7^ | Phe32 |  |  |  |  |  |  |
| **BF2*1501** | **cCD8α2** | **BF2*1501** | **cCD8α2** | **BF2*0401** | **cCD8α2** | **BF2*0401** | **cCD8α2** |
| Ile194 | Leu58 | Ile194 ^2^ | Arg60 | Ala218 | Asn104 | Ile194 ^2^ | Leu58, Arg60 |
| Val219 ^2^ | Asn104 | Ala218 | Asn104 | Val219 | Gln105 | Arg220 | Asn36 |
| Gln222 ^2^ | Tyr54 | Gln222 ^8^ | Ser39,Tyr54, Asn104 | Gln222 ^2^ | Tyr54, Asn102 | Gln222 | Tyr54 |
| Asp244 | Leu58 | Gln225 | Glu67 | Asp223 | Pro57 | Gln246 | Leu58 |
|  |  | Gln246 ^4^ | Leu58 |  |  |  |  |

The superscript number represent the number of val der Waals.

# Supplementary Figure

**Supplementary Figure 1.**


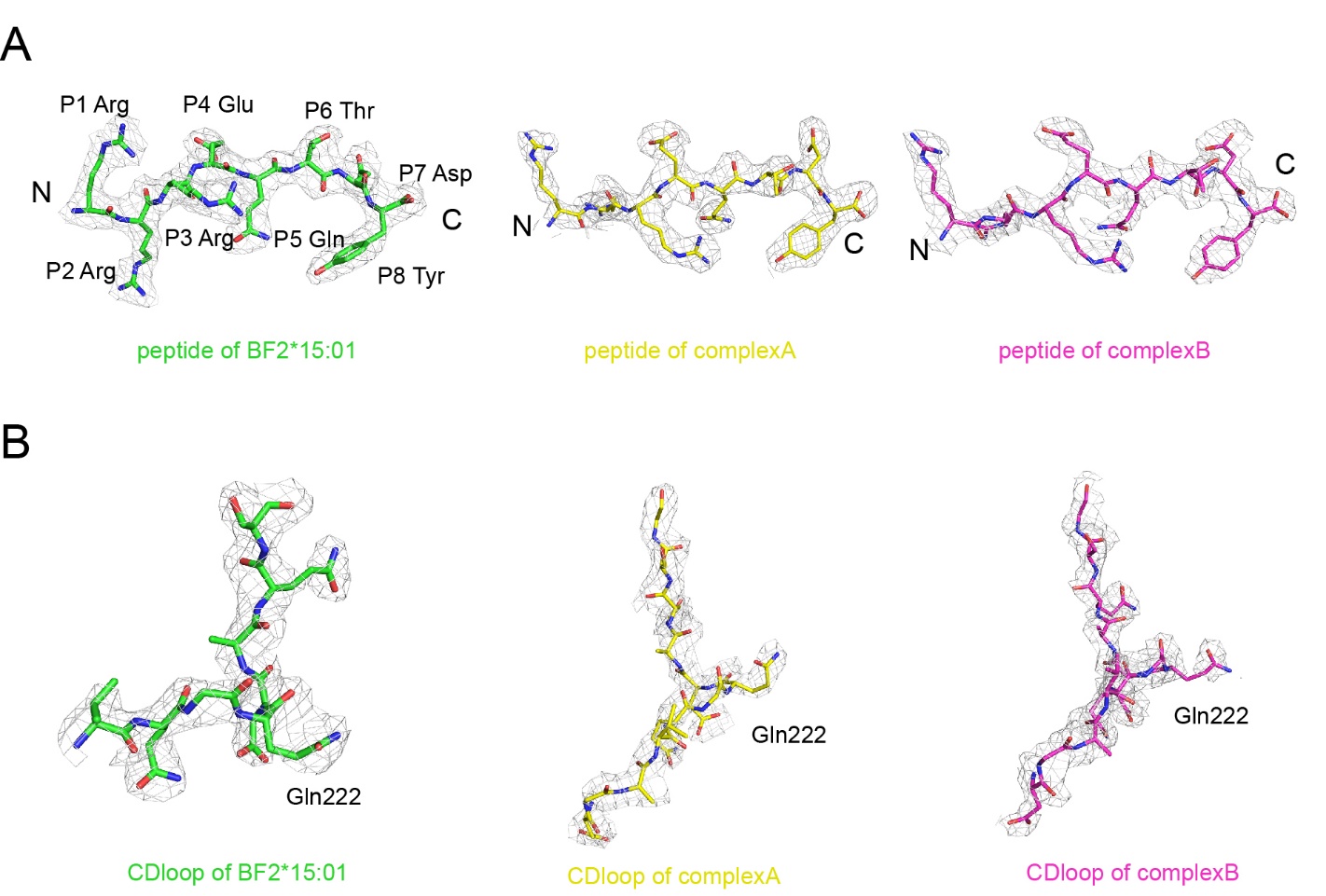


**Supplementary Figure 1. Electronic densities of RY0808 peptides and α3 domain CD loop of pBF2*1501, cCD8αα/pBF2*1501 and cCD8αα/pBF2*0401 complexes.**

**A**. Electron densities of RY0808 peptides of the pBF2*1501, cCD8αα/pBF2*1501 and cCD8αα/pBF2*0401 complexes. The peptides are colored green, yellow and pink, respectively. **B**. Electron densities of the α3 domain CD loop of the pBF2*1501, cCD8αα/pBF2*1501 and cCD8αα/pBF2*0401 complexes. The peptides are colored green, yellow and pink, respectively.
